# An Active Follow-up Strategy for Serological Suspects of Human African Trypanosomiasis with Negative Parasitology Set up by a Health Zone Team in the Democratic Republic of Congo

**DOI:** 10.3390/tropicalmed5020053

**Published:** 2020-04-04

**Authors:** Matthieu Nkieri, Florent Mbo, Papy Kavunga, Pathou Nganzobo, Titus Mafolo, Chalet Selego, Eric Mwamba Miaka

**Affiliations:** 1 Bagata Health Zone, Avenue Kalanganda N 10, Mwendo Bagata,32 Kwilu Province, Democratic Republic of the Congo; drmathieu.nkieri@gmail.com (M.N.); plukula@dndi.org (P.K.); 2 National Sleeping Sickness Control Program (PNLTHA) (PNMLS building), Boulevard Triomphale Crossing Av. 24 November, 10 Kinshasa, Democratic Republic of the Congo; drnganzobo@yahoo.fr (P.N.); erickmwamb2002@yahoo.fr (E.M.M.); 3 HAT Platform, Avenue Milambo N 4 Quartier Socimat, Gombe, 10 Kinshasa, Democratic Republic of the Congo; 4 Provincial Health Ministry of Kwilu, Aviation/Ifuri/Bandundu town, Bandundu, Democratic Republic of the Congo; titusmafolotitu@gmail.com (T.M.); selegochalenda2005@yahoo.fr (C.S.)

**Keywords:** CATT positive serological suspects, active follow-up strategy, human African trypanosomiasis

## Abstract

**Background:** The World Health Organization aims for the elimination of Human African Trypanosomiasis (HAT) as a public health problem by 2020 and for full elimination (absence of new cases) by 2030. One of strategies to achieve this is the active follow-up of all HAT serological suspects found during passive screening who have never been re-tested for parasitology. This is important because these cases can maintain HAT transmission and may be responsible for reemergence of the disease. **Methods:** In order to improve case finding at low cost in the targeted population, a general recall was transmitted to aparasitemic serological suspects about the availability of confirmation services at the general referral hospital. Transport was facilitated for re-testing. The initial examinations were carried out in Health Centers from Bagata Health Zone (HZ) in the Democratic Republic of the Congo between January 2017 and April 2019. This strategy of using a HZ team has not been previously documented. **Results:** From a total sample of 74 serological suspects listed by the health centers, 36 cases were re-examined at the general reference hospital; 19% (7/36) self-presented and 81% (29/36) were actively followed up by HZ personnel. Of those re-examined at the general reference hospital, 39% (14/36) resulted in a parasitologically confirmed case. Of the 14 people diagnosed with HAT, 14% (2/14) self-presented and the remaining 86% (12/14) were diagnosed in suspects who were actively followed up. This new strategy of facilitating transport from the villages added value by contributing to the detection of 12 HAT cases, compared to the passive approach, waiting for self-reference, which resulted in the detection of 2 new HAT cases. The cost per detected patient was 70 USD from the group of 7 suspects who self-presented for testing at the hospital and 346 USD per detected case for the group of 29 patients who were actively followed up by health zone staff. **Conclusion:** Targeted active follow-up of aparasitemic serological suspects by HZ teams is a cost-effective and promising approach to identifying additional cases of HAT in areas of very low prevalence, which would contribute to the HAT elimination goal set by the World Health Organization.

## 1. Background

WHO set the goal of eliminating HAT as a public health problem by 2020 and full elimination (absence of new cases) by 2030. To achieve elimination, all HAT cases will need to be detected using adapted strategies in the field. However, there are unexpected issues to resolve. One of these is tracing all HAT serological suspects who have never been tested for parasitology. Another is specifically targeting HAT serological suspects who had negative parasitology during active screening performed by traditional mobile teams and passive screening done at selected health facilities.

National sleeping sickness control programs (NSSCP) list, but rarely actively follow-up, subjects with positive serology but negative parasitology, who then remain untreated. Some will progress to the disease because the parasite was not detected for various reasons (including parasite fluctuation, technician performance, and lack of sensitive laboratory techniques).

There are two types of serological tests: The Card agglutination test for trypanosomiasis (CATT) and rapid diagnostic tests (RDT), which identify a large number of positive serological suspects. However, in order to positively define a HAT case and trigger treatment, parasitological confirmation is currently required.

In this article we focus on CATT positive serological suspects with negative parasitology, defined as aparasitemic serological suspects, which may be detected by two different approaches:

1. CATT positive aparasitemic serological suspects detected during active screening conducted by NSSCP mobile teams, which are unconfirmed despite using sensitive parasitological techniques (including mini anion exchange centrifugation technique (m-AECT)).

2. CATT positive aparasitemic serological suspects detected after spontaneously coming for passive screening conducted by health facilities with integrated HAT activities (which may include sensitive diagnostic techniques or only direct microscopy). This second group was the specific target of the activity.

General reference hospitals and reference health centers in endemic foci can perform sensitive diagnostic concentration techniques such as m-AECT or capillary tube centrifugation (CTC) but other health centers only perform microscopy on samples such as thick film, fresh blood, or lymph node puncture, which are less sensitive.

Usually, follow-up of CATT positive serological suspects with negative parasitology should be conducted by mobile teams during their annual active screening activities. During active screening in endemic villages, the mobile teams produce a report listing all aparasitemic serological suspects, which includes their addresses to facilitate follow-up.

These unconfirmed CATT positive serological suspects receive a recommendation during passive and active screening to go back to health facilities with HAT diagnostic capacity near their village every three months. Most suspects do not return to the recommended health facilities for follow-up because they are a long way from the village. They may not have the means to travel or may not feel very sick. During active screening, mobile teams can look for the remaining aparasitemic serological suspects from the previous year, but many of them do not participate in the screening visit for different reasons (absence from the village, fear of lumbar puncture, lack of confidentiality) [1].

According to the DRC Programme de Lutte contre la Trypanosomiase Humaine Africaine (National Sleeping Sickness control program, PNLTHA) data, over 20,000 aparasitemic serological suspects were reported in 2018 (unpublished data from the PNLTHA DRC, 2018 Annual report: Page 37, Table 5.2). In the three months before the beginning of the targeted active follow-up for passively detected aparasitemic serological suspects in Bagata health zone (HZ), no HAT patients had been diagnosed by Bagata mobile team and health centers.

Other authors have attempted to explain the number of HAT *T.b. gambiense* patients that remain undiagnosed. First, not all infected people are reached by screening activities [2]. Second, current diagnostic techniques do not pick up all *T.b. gambiense* infections due to lack of sensitivity of serological screening tests, of molecular techniques, or of the parasitological confirmation tests [3]. These undiagnosed, yet infected, people may act as a human reservoir of the parasite and might sustain transmission, forming a maintenance population [4]. Another potential human reservoir may be latent infections, also called ‘healthy carriers’, who do not always progress to clinical disease, although the relative contribution of these individuals to parasite transmission still needs to be documented. This last category was only recently described in Ivory Coast and consists of latently infected people that may carry trypanosomes for years or even decades [5].

In Guinea, patients with asymptomatic or latent infections were found to have consistently high titres in CATT/*T.b. gambiense* and positive results in the immune trypanolysis test, although no parasites could be detected in blood or lymph node fluid during a 2-year follow-up period [6]. This observation is in line with the fact that trypanosomes may survive in the extravascular spaces of diverse organs such as the heart, the central nervous system, and the skin [7,8]. Some projects conducted reactive searches using mobile teams to reach previously detected CATT or RDT positive serological suspects who had not responded to follow-up appointments for parasitology set up by mobile teams or health facilities.

Reactive searches have been implemented by specialized mobile teams and extended RDT detection centers supported by FIND (Foundation for Innovative New Diagnostics) tested with RDT 50 percent of HAT serological suspects (228/457) in the Province of Kongo Central in DRC between August 2015 and July 2016. Using parasitological methods, 111 cases were confirmed; this was 24 percent of all seropositive (111/457) or 49 percent of those examined (111/228) [9]. This is an important contribution to finding the remaining HAT cases, but an alternative simpler targeted approach could also contribute, such as active follow-up of aparasitemic serological suspects found during passive screening.

We describe here an alternative approach to reaching the aparasitemic serological suspects found during passive screening by permanent health staff that involves health workers and community health workers. After sensitization by health workers and community health workers, HZ teams used their means of transport (motorbike, car) to take all aparasitemic suspects, who had not previously independently returned, to the reference general hospital for follow-up appointments. This approach has not been used before.

## 2. Materials and Methods

### 2.1. Project Objective

To assess the added value of having HZ personnel actively following up and facilitating transport for aparasitemic serological suspects, who had been previously identified passively, for re-testing in order to improve case finding at low cost in a targeted population.

### 2.2. Strategy Used by the Health Zone Team

The aim of the HZ team was to organize active follow-up of aparasitemic serological suspects who would not return to the referral general hospital for re-testing by themselves after sensitization by health workers and community health workers. Most aparasitemic serological suspects found through passive screening do not respond to the follow-up appointments for re-testing, which are fixed at every three months according to PNLTHA recommendations. After three months, the aparasitemic serological suspect is considered as non-attendant. In our intervention, there was a two-week window after sensitization for all aparasitemic serological suspects to return spontaneously for re-testing to the referral general hospital. After this time, they were actively sought out by HZ staff.

### 2.3. Place of Work: Bagata Health Zone

The Bagata HZ, located at 535 km East of Kinshasa, in Kwilu province in the Democratic Republic of the Congo, is endemic for HAT. The Health Zone has eighteen health centers and one general reference hospital. Five of these health centers, and the general reference hospital, offer a package of HAT management activities: screening, diagnosis, and treatment. Six health facilities, including the general reference hospital, use CATT as the screening test; it is performed with fresh blood and not with serum. The general reference hospital diagnostic algorithm for serologically positive cases starts with lymph node palpation, and aspiration if enlarged cervical nodes are detected. The hospital performs sensitive diagnostic techniques such as m-AECT, modified simple centrifugation and CTC to search for HAT parasites. Parasite detection is confirmed under an LED microscope with a camera with 5 s video capture of the parasites for quality control. This general reference hospital was equipped as part of a clinical trial. The five health centers perform other less sensitive parasitological tests such as lymph node examination, and direct examination of thick blood film and fresh blood under the microscope.

### 2.4. Programmatic Follow-up of Activities

Before organizing this active follow-up approach, the HZ team retrospectively collected information about CATT positive serological suspects with negative parasitology from the five health centers and the referral general hospital described above from January 2017 to April 2019 using a PNLTHA data collection form. Suspects’ data were collected in register books as follows: initials of patient, age, address, sex, date of previous examination, signs and/or symptoms of HAT during the previous examination, CATT result, and results of any other parasitological diagnostic test. This initial data was completed, when available, with follow-up visits 1 and 2, including date, CATT result, diagnostic result, signs, and/or symptoms of HAT and the distance in kilometers between the home location of each aparasitemic serological suspect and the reference hospital. To the form we added additional information about whether the suspect reached the hospital by him- or herself or whether the hospital staff brought him/her with a car or motorbike. HZ teams started with sensitization sessions with health workers and community health workers during meetings at the five health centers with capacity to do serological testing. Community health workers then proceeded to locate the aparasitemic serological suspects to inform them about the importance of respecting their follow-up appointments. The HZ team mapped the home locations of CATT-positive serological suspects with negative parasitology, including a schedule for their active follow-up. Active follow-up by HZ staff of all aparasitemic serological suspects who had not spontaneously attended the recommended visits started after a two-week lag period. Bagata HZ vehicles were used to transport suspects who, after sensitization, were unable or unwilling to come by themselves to the reference general hospital. Transportation was also organized to return patients to their villages. Suspects living far from the referral hospital could stay overnight at the hospital without paying a fee. Food was provided at the hospital. Active follow-up of suspects was combined with supervisory visits by the HZ team to minimize costs, as the supervisory budget included accommodation and a per diem for health zone staff.

### 2.5. Ethical Aspects

Ethical approval was not needed as passive follow-up of aparasitemic serological suspects is included in the recommended routine activities of the PNLTHA in DRC.

### 2.6. Data Analysis

A simple descriptive analysis was performed. The target population is presented according to availability, showing the number and proportion of aparasitemic serological suspects re-tested, their sex and age, and whether they came spontaneously or were transported to the reference hospital; the parasitological results for all those re-tested are also mentioned. The institutional marginal cost data directly attributed to this additional activity were examined, without including the basic costs of provision of general health care. We included in the calculation: transport linked costs (fuel/oil + maintenance of the vehicles); training and motivation of the community health workers, direct supervision by the HZ staff food for the patients during their time in the hospital, and the cost of the m-AECT tests performed. No administrative costs or salaries have been considered as they would have existed anyway without the additional activity. Treatment was donated.

## 3. Results

### 3.1. Population Examined

In the lists gathered by Bagata HZ staff from 5 health centers and the reference hospital with capacity for HAT case detection, 74 persons were identified between January 2017 and April 2019 as aparasitemic serological suspects (Table S1). Of these, 36 were found and tested using parasitological techniques. The small number of detected individuals that could be effectively followed up (36/74, 49% of suspects) shows the limits of this case search process with the available funds.

**Figure 1:**
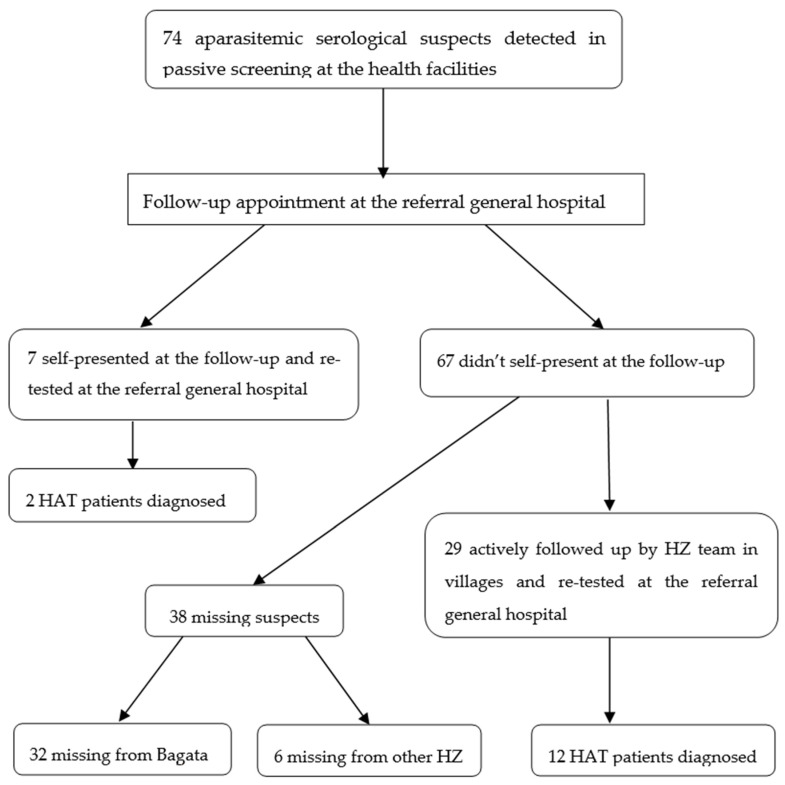
Flow diagram including passive approach (spontaneous visits after sensitization) and active follow-up strategy.

The average age was 30 years for selected suspects and 34 for detected cases; 54% (40/74) of suspects and 36% (5/14) of detected cases were female. 9% (7/74) travelled to the hospital by their own means for follow-up examinations and 39% (29/74) were actively recovered by the HZ team, see Figure 1.

51% (38/74) of aparasitemic serological suspects were not followed by HZ staff for re-testing and remained untested (Table 1). Of these, 6 had been detected in Bagata, but lived in other HZ.

### 3.2. Cases Detected

Of the aparasitemic serological suspects who self-presented (passive approach), 14% (2/14) were diagnosed with HAT. The remaining 86% of all confirmed HAT cases (12/14) were diagnosed in suspects who HZ personnel actively followed up. Overall, 39% (14/36) of CATT-positive suspects with negative parasitology coming from health centers were confirmed positive with sensitive diagnostic techniques such as m-AECT, CTC and modified simple centrifugation performed in the reference general hospital, although two of them required a second visit (Table S2). Two confirmed cases had a negative CATT test during the first visit, one was confirmed after a second positive CATT test in a supplementary visit and the other may have been a manipulation error and was confirmed in the parasitological exam at the same visit when the negative serology was found, and 42% (6/14) of new HAT cases were in the first stage of the disease and 58% (8/14) in the second stage. The positive predictive value of the screening test increased to 52% for the 36 CATT positive suspects with negative parasitology who responded to one or two follow-up visits at the reference general hospital for parasitology examination. Of the CATT positive suspects with negative parasitology who were followed, 30% (11/36) were found to be CATT negative during the follow-up visit at the reference general hospital.

### 3.3. Cost Assessment of the Strategy

We calculated the additional institutional cost to organize and execute the new activities.

The active follow-up strategy implemented by the HZ team had an additional cost of 306 USD per new case detected, taking into consideration all 14 new cases, including those that came spontaneously after the community health workers’ sensitization visits (Table 2). Dividing the attributable costs between the patients that came spontaneously (their proportion of food, sensitization, and m-AECT) and those that had to be transported (adding all transport and supervision costs to this group), the cost per detected patient is 70 USD from the group of 7 suspects who self-presented for testing at the hospital and 346 USD per detected case for the group of 29 patients who were actively followed up by health zone staff.

## 4. Discussion

Despite the fact that almost half of the identified suspects missed the follow-up examination and possible confirmation by sensitive parasitological testing, these data show that this new active follow-up strategy improved detection of HAT cases. It also shows that with the existing economic means at the time of the investigation, the passive approach will have a limited impact on the interruption of HAT transmission up to 2030. Four suspects that self-referred to the health structure and nine of those examined via the active follow-up strategy, who remained CATT positive, could not be confirmed. These 13 cases, along with the 38 missing individuals, fall into the category of unconfirmed suspects who may contribute to re-emergence of the disease. The HZ staff should continue to follow them until their serology becomes negative or parasites are detected. The present strategy, while only covering a single HZ and a limited number of seropositive suspects, seems to be cost effective as it targets a high-risk subgroup of the population. We believe that to maximize the detection of HAT cases, this strategy should be generalized and that it must be combined with a continued extension of serological testing and follow-up of positive cases in known endemic areas.

In Uganda, in a low prevalence environment, Lee and Palmer discovered the limitations of a surveillance strategy that used RDT to progress to the next step of confirming serological suspects. Patient misunderstanding of the referral rationale was a key structural weakness due to poor provider communication about the possibility of discordant HAT test results. The biggest difficulty was how to communicate the concept of possible false positives in the serological test and the need to find parasites microscopically in order to be able to treat; this may lead the patients to distrust the whole system. Transport and possible health services costs were an additional deterrent [10].

The high proportion of new cases detected corroborates the usefulness of sensitive techniques, as described by Robays et al., 2004 [2], who examined the diagnostic process used in active screening performed by mobile teams in the field in DRC stating that, once a sensitive algorithm is in place, the attendance rate is a critical factor.

Pepin et al., 1989 [11], showed that passive screening followed by an active follow-up strategy at health facilities level is likely to improve case detection. The same authors also showed that in the Nioki health zone, Democratic Republic of the Congo in 1987 health centers and the general referral hospital could detect 71.5% of all patients after re-training personnel and updating equipment.

Penchenier et al., 1991, reported that 30.6% of 193 suspected positives to either blood or serum (discordant) became negative after one month, mentioning the possibility of false positivity or cross-reaction problems, with another cause being blood hemagglutination, and pointing at possible contact with animal trypanosomes in case of a previously positive serum sample [12]. In our sample we found 10/36 (28 percent) became negative for CATT in blood and parasitology.

The active follow-up strategy of previously CATT positive parasitologically unconfirmed suspects in our analysis in Bagata allowed the identification of 43% (6/14) of new cases in the early stage of the disease, thus breaking the transmission chain and stopping the evolution towards the advanced stage.

By comparison, an analysis in a context with higher prevalence by Lutumba et al., 2007, showed that the best cost-effectiveness of a confirmation strategy after screening with CATT was the combination with lymph node puncture and two concentration methods (CTC and m-AECT). It was calculated as 265.46€ per life saved. [13]. The costs of performing the two tests (m-AECT and CTC) of the current study in the field have not been elucidated yet. The cost per patient detected would increase if more suspects were searched via an active follow-up strategy by motorbike or vehicle, as those not examined tended to live further away and were more difficult to find. In a low prevalence context, the active follow-up strategy would be more efficient compared with traditional active screening performed by mobile teams; Bagata mobile teams did not detect any HAT cases in the same area for the three months before starting this strategy.

The remaining CATT positive suspects with negative parasitology after screening at the hospital may suggest, according to other authors, the existence of non-virulent or non-pathogenic trypanosome strains and/or human susceptibility that may lead to long-term seropositivity without detectable parasitemia [14]. This may also suggest the possibility or existence of a human reservoir of trypanosomes that can contribute to the maintenance or periodic resurgence of HAT in endemic foci, often attributed to an animal reservoir [15,16,17,18].

A recent cost analysis has shown a disproportionate increase in the cost per case detected by mobile teams using traditional methods, which is directly related to the reduction in prevalence. According to this analysis, the average cost per case detected for 10 mobile teams in DRC climbed from 1,789 USD in 2014 to 21,324 USD in 2018 [19]. Mini mobile teams acting door to door resulted in a lower cost per individual examined (1.67 vs 1.89) when compared with the full mobile teams, but screening the general population still cost 110,994 USD per mini mobile team and 122,932 per traditional mobile team in DRC per year [20]. To improve the effectiveness of the active search strategy, a door-to-door method has been the targeted option, which involves looking for previously identified cases or seropositive individuals and people in their immediate environment [21].

These observations indicate that we must combine several strategies, adapted to the local epidemiology, to maintain cost-effectiveness [22]. They need to include active follow-up of suspects in a low prevalence context in order to detect the last cases of sleeping sickness.

An active follow-up strategy can be extended to the remaining CATT or RDT positive suspects with negative parasitological results found during active screening, who were not considered for this investigation. Health facilities that are able to perform serological testing would need to be included in a network with the health centers and general reference hospitals able to perform sensitive diagnostic tests. To make this network functional, several aspects need to be considered and developed: training of laboratory technicians, a regular supply of laboratory equipment and consumables, sensitization of community agents and health workers, and transport of serologically positive suspects.

## 5. Conclusions

An active follow-up strategy that targets CATT-positive or RDT-positive serological suspects by implementing a HZ team would be a cost-effective and promising approach to identifying the last cases of HAT in areas of very low prevalence, which would contribute to the HAT elimination goal set by the World Health Organization. The integration of this strategy in the activities of the general health system would allow for sustainability.

## Figures and Tables

**Table 1:** Achievements of passive and active follow-up approaches with parasitology results.

Category of Suspects	N	CATT Result at Follow-Up	Parasitology Result at Follow-Up
Suspects who self-presented	7	6 positives 1 negative	2 HAT patients
Suspects actively followed-up	29	19 positives 10 negatives	11 HAT patients 1 HAT patient
Suspects not re-examined	38	N/A	N/A

N/A: Not available.

**Table 2:** Marginal direct cost of active search strategy from January 2017 to April 2019.

Materials Activities	Quantity	Unit Cost (USD)	Total Cost (USD)	Observation
Diesel + Gasoline + Oil	800 + 420 + 23 L	1.5/1.8/5	2071	Transportation and river crossing
Maintenance (vehicle and motorbike)	-	-	900	-
Supervision cost	-	-	600	Health zone team
Food for the suspects	36	10	360	Hospital stay
Sensitization of Community agents	50	5	250	Location of suspects
m-AECT	36	3	108	-
Total	-	-	4289	-

## Supplementary Materials

The following are available at https://www.mdpi.com/2414-6366/5/2/53/s1. Table S1: Number of CATT positive serological suspects collected in Bagata Health Zone (HZ), Table S2: Signs and test results of 36 CATT positive serological suspects at previous exam at health centers and at the follow-up visits at the hospital.
