# Supplementary material for: An Active Follow-up Strategy for Serological Suspects of Human African Trypanosomiasis with Negative Parasitology Set up by a Health Zone Team in the Democratic Republic of Congo
Source: Trop Med Infect Dis. 2020 Apr 4;5(2):53. doi: 10.3390/tropicalmed5020053 (PMC7345707; doi:10.3390/tropicalmed5020053)
Supplement: Supplementary file 1 [file tropicalmed-05-00053-s001.pdf]

Tropical Medicine and Infectious Disease

MDPI

Supplementary Materials

An Active Follow-up Strategy for Serological Suspects of Human African Trypanosomiasis with Negative Parasitology Set up by a Health Zone Team in the Democratic Republic of Congo

Matthieu Nkieri [1,] †, Florent Mbo [2,3,] †,*, Papy Kavunga [1], Pathou Nganzobo [2], Titus Mafolo [4], Chalet Selego [4] and Eric Mwamba Miaka [2]

1 Bagata Health Zone, Avenue Kalanganda N 10, Mwendo Bagata,32 Kwilu province, Democratic Republic of Congo; drmathieu.nkieri@gmail.com (M.N.); plukula@dndi.org (P.K.)

2 National Sleeping Sickness Control Program (PNLTHA) (PNMLS building), Boulevard Triomphale crossing Av. 24 November, 10 Kinshasa, Democratic Republic of Congo; drnganzobo@yahoo.fr (P.N.); erickmwamb2002@yahoo.fr (E.M.M.)

3 HAT Platform, Avenue Milambo N 4 Quartier Socimat, Gombe ,10 Kinshasa , Democratic Republic of Congo(F.M)

4 Provincial Health Ministry of Kwilu, Aviation/Ifuri/ Bandundu town, Democratic Republic of Congo; titusmafolotitu@gmail.com (T.M.); selegochalenda2005@yahoo.fr (C.S.)

† These authors contributed equally

* Correspondence: fmbo@dndi.org; docflorentmbo@yahoo.fr; Tel.: +243814313838

Received: 6 January 2020; Accepted: 2 April 2020; Published: date

Table S1. Number of CATT positive serological suspects collected in Bagata Health Zone (HZ).

Suspect Number	Sex	Age	HZ	Previous Exam at HC	CATT	HAT Diagnosis at HC	Main Signs and Symptoms	Distance Location to Hospital	Arrival to Hospital	Follow-Up by Reactive Search
1	F	31	BGTA	01.09.2017	pos	neg.	headache	NA	no	NA
2	F	29	BGTA	28.10.2017	pos	neg.	headache, dizziness	NA	no	NA
3	M	11	BGTA	27.12.2017	pos	neg.	headache, backpain	NA	no	NA
4	F	41	BGTA	09.01.2017	pos	neg.	headache	1	herself	no
5	M	9	BGTA	24.01.2018	pos	neg.	headache	NA	no	NA

6	F	23	BGTA	04.01.2018	pos	neg.	back pain	1	herself	no
7	M	47	BGTA	01.03.2017	pos	neg.	pruritus	NA	no	NA
8	M	28	BGTA	13.03.2017	pos	neg.	nausea	1	himself	no
9	F	37	BGTA	30.04.2018	pos	neg.	headache	1	herself	no
10	F	60	BGTA	25.12.2017	pos	neg.	headache	1	herself	no
11	F	7	BGTA	31.01.2018	pos	neg.	nausea	NA	no	NA
12	F	32	BGTA	27.05.2018	pos	neg.	pruritus	1	Motorbike	yes
13	M	13	BGTA	26.06.2018	pos	neg.	pruritus	20	Motorbike	yes
14	M	19	BGTA	16.11.2017	pos	neg.	fever	NA	no	NA
15	M	65	BGTA	15.12.2017	pos	neg.	anorexia	20	Motorbike	yes
16	M	15	BGTA	15.12.2017	pos	neg.	headache	20	Motorbike	yes
17	F	57	BGTA	16.11.2017	pos	neg.	sleepiness	20	Motorbike	yes
18	M	45	BGTA	10.013.2017	pos	neg.	headache, fever	NA	no	NA
19	M	47	BGTA	15.04.2017	pos	neg.	nausea	37	Motorbike	yes
20	M	68	BGTA	24.08.2018	pos	neg.	nausea	NA	no	NA
21	M	34	BGTA	24.08.2018	pos	neg.	fever	NA	no	NA
22	F	29	BGTA	24.08.2018	pos	neg.	headache	68	Motorbike	yes
23	M	31	BGTA	24.08.2018	pos	neg.	nausea	76	Vehicle	yes
24	F	24	BGTA	26.06.2019	pos	neg.	nausea	59	Vehicle	yes
25	M	13	BGTA	20.09.2018	pos	neg.	pruritus	10	Motorbike	yes
26	F	33	BGTA	09.07.2017	pos	neg.	fever	17	Motorbike	yes
27	M	19	BGTA	07.09.2017	pos	neg.	pruritus	10	Motorbike	yes
28	F	3	BGTA	07.09.2017	pos	neg.	fever	17	Motorbike	yes
29	M	19	BGTA	07.09.2017	pos	neg.	sleepiness	10	himself	no
30	M	19	BGTA	07.08.2017	pos	neg.	sleepiness	10	himself	no
31	F	46	BGTA	20.12.2017	pos	neg.	fever	120	Vehicle	yes
32	M	56	BGTA	20.12.2017	pos	neg.	headache	NA	no	NA
33	F	25	BGTA	12.102018	pos	neg.	nausea	122	Vehicle	yes
34	F	23	BGTA	11.10.2018	pos	neg.	sleepiness	84	Vehicle	yes
35	F	20	BGTA	23.11.2017	pos	neg.	nausea	84	Vehicle	yes
36	M	5	BGTA	04.07.2019	pos	neg.	fever	NA	no	NA

37	F	5	BGTA	04.07.2019	pos	neg.	fever	NA	no	NA
38	F	29	BGTA	04.07.2019	pos	neg.	pruritus	NA	no	NA
39	F	54	SIA	27.05.2018	pos	neg.	anorexia	NA	no	NA
40	M	26	SIA	09.06.2018	pos	neg.	headache	NA	no	NA
41	F	15	SIA	17.05.2019	pos	neg.	pruritus	NA	no	NA
42	M	25	SIA	17.05.2019	pos	neg.	anorexia	NA	no	NA
43	F	10	KKGO	30.03.2019	pos	neg.	headache	NA	no	NA
44	M	34	DJUMA	04.02.2019	pos	neg.	pruritus	NA	no	NA
45	M	33	BGTA	04.09.2017	pos	neg.	anorexia	90	Motorbike	yes
46	F	24	BGTA	9.09.2017	pos	neg.	Fever	90	Motorbike	yes
47	F	26	BGTA	23.09.2017	pos	neg.	Nausea	NA	no	NA
48	F	44	BGTA	27.092017	pos	neg.	insomnia	NA	no	NA
49	F	36	BGTA	04.11.2017	pos	neg.	insomnia	90	Motorbike	yes
50	F	7	BGTA	04.11.2017	pos	neg.	sleepiness	NA	no	NA
51	M	43	BGTA	11.12.2017	pos	neg.	headache	NA	no	NA
52	M	10	BGTA	05.01.2018	pos	neg.	fever	NA	no	NA
53	M	34	BGTA	10.01.2018	pos	neg.	insomnia	NA	no	NA
54	F	41	BGTA	03.02.2018	pos	neg.	fever	NA	no	NA
55	M	46	BGTA	09.12.2018	pos	neg.	pruritus	104	Vehicle	yes
56	M	39	BGTA	04.07.2018	pos	neg.	headache	87	Vehicle	yes
57	M	41	BGTA	07.07.2018	pos	neg.	nausea	96	Vehicle	yes
58	M	21	BGTA	12.08.2018	pos	neg.	headache	NA	no	NA
59	F	6	BGTA	19.08.2018	pos	neg.	fever	NA	no	NA
60	F	24	BGTA	19.08.2018	pos	neg.	nausea	NA	no	NA
61	F	30	BGTA	19.09.2017	pos	neg.	fever	67	Motorbike	yes
62	M	42	BGTA	10.02.2018	pos	neg.	anorexia	20	Motorbike	yes
63	F	54	BGTA	17.02.2018	pos	neg.	fever	20	Motorbike	yes
64	M	28	BGTA	25.02.2018	pos	neg.	nausea	NA	no	NA
65	F	34	BGTA	25.02.2018	pos	neg.	fever	NA	no	NA
66	F	31	BGTA	04.03.2018	pos	neg.	headache	NA	no	NA
67	F	16	BGTA	23.05.2018	pos	neg.	anorexia	NA	no	NA

68	F	59	BGTA	04.06.2018	pos	neg.	fever	36	Motorbike	yes
69	F	40	BGTA	02.12.2017	pos	neg.	pruritus	NA	no	NA
70	F	32	BGTA	08.01.2018	pos	neg.	anorexia	NA	no	NA
71	F	21	BGTA	03.04.2018	pos	neg.	fever	59	Vehicle	yes
72	M	31	BGTA	15.03.2018	pos	neg.	anorexia	64	Vehicle	yes
73	M	38	BGTA	09.08.2018	pos	neg.	nausea	NA	no	NA
74	F	22	BGTA	21.08.2018	pos	neg.	asthenia	NA	no	NA

Table S2. Signs and test results of 36 CATT positive serological suspects at previous exam at health centers and at the follow-up visits at the hospital.

Suspect Number	Sex	Age	Initial Test at HC	CATT	Signs	Follow up 1	CATT	HAT Diagnosis at Hospital	Signs	Follow up 2	CATT	HAT Diagnosis at Hospital	Signs
1	F	41	09.01.2017	pos	headache	24.04.2019	neg	neg	headache	no			
2	F	23	04.01.2018	pos	back pain	24.04.2018	pos	neg	back pain	no			
3	M	28	13.03.2017	pos	nausea	13.06.2017	pos	neg	nausea	no			
4	F	37	30.04.2018	pos	headache	30.08.2018	pos	neg	nausea	no			
5	F	60	25.12.2017	pos	headache	25.03.2018	pos	pos	dizziness	no			
6	F	32	27.05.2018	pos	pruritus	27.08.2018	neg	neg	nausea	no			
7	M	13	26.06.2018	pos	pruritus	12.09.2018	pos	pos	fever	no			
8	M	65	15.12.2017	pos	anorexia	21.03.2018	pos	pos	nausea	no			
9	M	15	15.12.2017	pos	headache	21.03.2018	pos	pos	diarrhea	no			
10	F	57	16.11.2017	pos	sleepiness	16.02.2018	pos	neg	nausea	no			
11	M	47	15.04.2017	pos	nausea	15.07.2017	pos	neg	nausea	no			
12	F	29	24.08.2018	pos	headache	24.11.2018	pos	neg	back pain	no			
13	M	31	24.08.2018	pos	nausea	24.11.2018	pos	neg	diarrhea	24.03.2019	pos	pos	headache
14	F	24	26.06.2019	pos	nausea	28.09.2019	neg	neg	headache	no			
15	M	13	20.09.2018	pos	pruritus	20.12 2018	pos	pos	pruritus, headache	no			
16	F	33	09.07.2017	pos	fever	07.12.2017	pos	neg	nausea	no			
17	M	19	07.09.2017	pos	pruritus	07.12.2017	neg	neg	nausea	12.03.2019	pos	pos	fever
18	F	3	07.09.2017	pos	fever	07.12.2017	neg	neg	fever	no			
19	M	19	07.09.2017	pos	sleepiness	07.12.2018	pos	neg	fever	no			

20	M	19	07.08.2017	pos	sleepiness	16.11.2017	pos	pos	sleepiness	no
21	F	46	20.12.2017	pos	fever	20.03.2018	neg	neg	fever	no
22	F	23	11.10.2018	pos	sleepiness	11.02.2019	neg	pos	sleepiness, pruritus	no
23	F	20	23.11.2017	pos	nausea	28.02.2018	pos	neg	fever	no
24	M	33	04.09.2017	pos	anorexia	12.12.2018	pos	neg	nausea	no
25	F	24	09.09.2017	pos	fever	10.12.2017	neg	neg	fever	no
26	F	36	04.11.2017	pos	insomnia	06.02.2018	pos	neg	sleepiness	no
27	M	46	09.12.2018	pos	pruritus	12.05.2019	pos	pos	pruritus, fever	no
28	M	39	04.07.2018	pos	headache	06.10.2018	pos	neg	fever	no
29	M	41	07.07.2018	pos	nausea	08.10.2018	neg	neg	pruritus	no
30	F	30	19.09.2017	pos	fever	19.12.2017	pos	pos	fever	no
31	M	42	10.02.2018	pos	anorexia	12.05.2019	neg	neg	pruritus	no
32	F	54	17.02.2018	pos	fever	22.03.2019	pos	pos	anorexia	no
33	F	25	25.02.2018	pos	nausea	26.05.2018	neg	neg	headache	no
34	F	59	04.06.2018	pos	fever	06.09.2019	pos	pos	anorexia, fever	no
35	F	21	03.04.2018	pos	fever	04.07.2018	pos	neg	pruritus	no
36	M	31	15.03.2018	pos	anorexia	15.06.2018	pos	pos	insomnia	no
